# Relapses and serious adverse events during rituximab maintenance therapy in ANCA-associated vasculitis: a multicentre retrospective study

**DOI:** 10.1093/rheumatology/keae409

**Published:** 2024-08-06

**Authors:** Chrysoula G Gialouri, Aglaia Chalkia, Christos Koutsianas, Katerina Chavatza, Evangelia Argyriou, Alexandros Panagiotopoulos, Anastasios Karamanakos, Aikaterini Dimouli, Christina Tsalapaki, Konstantinos Thomas, Philippos Orfanos, Pagona Lagiou, George Katsikas, Kyriaki Boki, Dimitrios Boumpas, Dimitrios Petras, Dimitrios Vassilopoulos

**Affiliations:** Joint Rheumatology Program, Clinical Immunology-Rheumatology Unit, 2nd Department of Medicine and Laboratory, National and Kapodistrian University of Athens, School of Medicine, General Hospital of Athens “Hippokration”, Athens, Greece; Department of Hygiene, Epidemiology and Medical Statistics, School of Medicine, National and Kapodistrian University of Athens, Athens, Greece; Nephrology Department, General Hospital of Athens “Hippokration”, Athens, Greece; Joint Rheumatology Program, Clinical Immunology-Rheumatology Unit, 2nd Department of Medicine and Laboratory, National and Kapodistrian University of Athens, School of Medicine, General Hospital of Athens “Hippokration”, Athens, Greece; Joint Rheumatology Program, Clinical Immunology-Rheumatology Unit, 4th Department of Medicine, National and Kapodistrian University of Athens School of Medicine, Attikon General Hospital, Athens, Greece; Rheumatology Unit, Sismanoglio General Hospital, Athens, Greece; Joint Rheumatology Program, Clinical Immunology-Rheumatology Unit, 2nd Department of Medicine and Laboratory, National and Kapodistrian University of Athens, School of Medicine, General Hospital of Athens “Hippokration”, Athens, Greece; Department of Rheumatology, “Evangelismos” General Hospital, Athens, Greece; Department of Rheumatology, “Evangelismos” General Hospital, Athens, Greece; Joint Rheumatology Program, Clinical Immunology-Rheumatology Unit, 2nd Department of Medicine and Laboratory, National and Kapodistrian University of Athens, School of Medicine, General Hospital of Athens “Hippokration”, Athens, Greece; Joint Rheumatology Program, Clinical Immunology-Rheumatology Unit, 4th Department of Medicine, National and Kapodistrian University of Athens School of Medicine, Attikon General Hospital, Athens, Greece; Department of Hygiene, Epidemiology and Medical Statistics, School of Medicine, National and Kapodistrian University of Athens, Athens, Greece; Department of Hygiene, Epidemiology and Medical Statistics, School of Medicine, National and Kapodistrian University of Athens, Athens, Greece; Department of Rheumatology, “Evangelismos” General Hospital, Athens, Greece; Rheumatology Unit, Sismanoglio General Hospital, Athens, Greece; Joint Rheumatology Program, Clinical Immunology-Rheumatology Unit, 4th Department of Medicine, National and Kapodistrian University of Athens School of Medicine, Attikon General Hospital, Athens, Greece; Nephrology Department, General Hospital of Athens “Hippokration”, Athens, Greece; Joint Rheumatology Program, Clinical Immunology-Rheumatology Unit, 2nd Department of Medicine and Laboratory, National and Kapodistrian University of Athens, School of Medicine, General Hospital of Athens “Hippokration”, Athens, Greece

**Keywords:** ANCA-associated vasculitis, rituximab, maintenance, relapse, infections

## Abstract

**Objectives:**

There are limited real-life data regarding the efficacy and safety of rituximab (RTX) as a remission maintenance agent in microscopic polyangiitis (MPA) and granulomatosis-with-polyangiitis (GPA). We aimed to estimate the incidence and risk factors for relapses, as well for serious adverse events (SAEs) in MPA/GPA patients during RTX maintenance.

**Methods:**

A retrospective cohort of newly diagnosed/relapsing GPA/MPA patients who received RTX maintenance (≥1 RTX cycle, ≥6 months follow-up) following complete remission (BVAS version-3 = 0 plus prednisolone ≤7.5 mg/day) with induction regimens. SAEs included serious infections, COronaVIrus-Disease 2019 (COVID-19)–associated hospitalizations, deaths, cardiovascular events, malignancies and hypogammaglobulinemia. The incidence rates (IRs) and relapse-free survival were estimated through Kaplan–Meier plots. Cox regression was conducted to investigate factors associated with the time-to-relapse.

**Results:**

A total of 101 patients were included: 48% females, 69% GPA, 53% newly diagnosed, median age 63 years. During follow-up (294.5 patient-years, median: 3 RTX cycles), 30 relapses (57% major) occurred among 24 patients (24%, IR 10.2/100 patient-years). Kidney involvement (adjusted hazard ratio/aHR: 0.20; 95% CI: 0.06–0.74, *P* = 0.016), prior induction with RTX plus CYC (*vs* RTX monotherapy: aHR = 0.02; 95% CI: 0.001–0.43, *P = *0.012) and shorter time interval until complete remission (aHR = 1.07; 95% CI: 1.01–1.14, *P = *0.023) were associated with decreased relapse risk. We recorded 17 serious infections (IR 5.8/100 patient-years), 11 COVID-19–associated hospitalizations (IR 3.7/100 patient-years), 4 malignancies (IR 1.4/100 patient-years), 6 cardiovascular events (IR 2/100 patient-years) and 10 deaths (IR 3.4/100 patient-years).

**Conclusion:**

In this real-world study, relapses during RTX maintenance occurred in approximately 1 out of 4 patients. Kidney involvement, induction with RTX plus CYC, and earlier achievement of complete remission were associated with lower relapse risk. The serious infections rate was consistent with previous reports, whereas an increased rate of COVID-19–associated hospitalizations was observed.

Rheumatology key messages

- Relapses in GPA/MPA during RTX maintenance occur in ∼25% of patients, usually within the first 2 years.
- Kidney involvement, induction with rituximab plus cyclophosphamide, and earlier complete remission are associated with lower relapse risk.
- Serious infections remain a concern for GPA/MPA patients during RTX maintenance, constituting the leading cause of death.

## Introduction

Granulomatosis with polyangiitis (GPA) and microscopic polyangiitis (ΜPA) are subgroups of ANCA-associated vasculitides (AAV) characterized by necrotizing inflammation of small to medium vessels in multiple organs with vasculitic and/or granulomatous manifestations [1]. The advancements in therapeutic strategies for GPA and MPA have improved their short-term prognosis, transforming them from acute and life-threatening, to chronic diseases with a remitting–relapsing course [2, 3].

Despite the successful control of active disease with induction regimens [4], up to 50% of AAV patients will relapse, even during maintenance therapy [5]. Relapses may require further exposure to glucocorticoids (GCs) and prolonged immunosuppressive medication, which in turn contribute to toxicity and infection risk [6], leading to accruing disease-/treatment-related damage [7]. Evidence from heterogeneous AAV cohorts suggests various predictors of relapse, such as serological (PR3-ANCA positivity or ANCA-levels rise) and clinical (GPA, better kidney function) features. Additionally, the agents used to induce and/or maintain remission and the duration of remission maintenance therapy have been associated with the relapse risk [8].

Rituximab (RTX) is an anti-CD20 monoclonal antibody that depletes autoreactive B cells, which are crucial mediators in AAV pathogenesis, but not bone marrow B cell precursors and long-lived plasma cells in inflamed tissues, which probably explains the repopulation of the peripheral B cell compartment and the persistent Ig production. RTX has been proven non-inferior to CYC for remission induction in GPA/MPA [9, 10], and is now recommended as the first choice for remission maintenance [11]. Real-life data assessing the efficacy and safety of RTX maintenance therapy are limited [12–15].

Herein, we aimed to estimate the incidence and risk factors for relapses, as well for serious adverse events (SAEs) during RTX maintenance in GPA/MPA patients after complete remission has been achieved.

## Methods

### Patient population, data collection and study design

We conducted a retrospective cohort study of patients with newly diagnosed or relapsing GPA/MPA (Chapel Hill Consensus Conference definitions [16]), consecutively followed in four rheumatology/nephrology clinics as part of the Greek AAV Patient Registry [17] (06/2008–09/2023). The study was approved by the Institutional Review Board of the General Hospital of Athens “Hippokration” (Scientific Council number: 9546/19–05-2023). Written informed consent was obtained from all participants.

Patients with GPA/MPA who were in complete remission (defined as BVAS version 3 (BVASv3)=0 and a prednisolone equivalent dose of ≤7.5 mg/day) following induction, who had received ≥1 RTX cycle (ie 6-monthly infusions) as remission maintenance therapy and who had been followed for ≥6 months were included. RTX was administered in a fixed 6-month schedule, while the dose was at the physicians’ discretion (500–2000 mg per cycle).

The follow-up was computed from RTX maintenance initiation (date of first RTX cycle under complete remission) to: (i) relapse, death, RTX discontinuation due to a side effect, or switch of maintenance agent for other reasons; (ii) last RTX cycle plus 6 months or (iii) last patient contact. Each patient could contribute to the overall follow-up with >1 RTX maintenance course (i.e. total duration until RTX maintenance discontinuation), following a relapse or delayed cycle. For each course, the cause of the last RTX cycle was recorded: end of study/last visit, physician’s decision for maintenance discontinuation, relapse, death, side effect (e.g. infusion-related, hypogammaglobulinemia), non-compliance with therapy, delayed cycle [>6 months; cases within the COronaVIrus-Disease 2019 (COVID-19) pandemic, physician’s decision for 1-year interval between RTX cycles, etc.] or lost to follow-up (LFU). The study design is shown in Supplementary Fig. S1, available at *Rheumatology* online.

### Relapses and serious adverse events

Relapse was defined [5] as any increase in BVASv3 > 0, with major relapses involving ≥1 major/vital organ, a life-threatening manifestation, or both, necessitating new induction therapy. Minor relapses were defined as those not corresponding to major, but requiring mild treatment intensification, such as i.m. GCs or an incremental increase in the oral GC dose, addition of a second agent (such as MTX, AZA or MMF) or continuation of RTX maintenance therapy as was scheduled without additional interventions.

SAEs during follow-up included: (1) serious infections [SIs; requiring hospitalization (excluding those associated with SARS-COV-2) or i.v. antibiotics and opportunistic infections, including herpes zoster); (2) COronaVIrus-Disease 2019 (COVID-19)–associated hospitalizations; (3) deaths; (4) cardiovascular events (CVEs; hospitalization for heart failure, coronary revascularization, myocardial infarction, stroke, arterial/venous thrombosis, peripheral artery revascularization); (5) malignancies; and (6) hypogammaglobulinemia [IgG < 700 mg/dl; mild (699–500 mg/dl), moderate (499–300 mg/dl) or severe (<300 mg/dl)].

We recorded: (i) demographic and disease-related characteristics: sex, age at RTX maintenance initiation, AAV phenotype (GPA/MPA), ANCA serotype ever present (c-/PR3-ANCA, p-/MPO-ANCA positivity or ANCA-negativity), disease duration (time interval from diagnosis to RTX maintenance initiation), previous relapses; (ii) induction treatment received until complete remission: RTX monotherapy or combined with other immunosuppressives (MTX, MMF), CYC monotherapy, RTX + CYC combination; (iii) need for haemodialysis or/and plasma exchange (PLEX); (iv) organ involvement (according to BVASv3 components) and disease activity (BVASv3) [18, 19]; (v) comorbidities (definitions in Supplementary Data S1, available at *Rheumatology* online): hypertension, diabetes, history of cardiovascular disease and/or stroke, depression, osteoporotic fractures, peptic ulcer or other stomach problems, chronic obstructive pulmonary disease and cancer history, also calculating the Rheumatic Disease Comorbidity Index [20]; (vi) disease-related damage at RTX maintenance initiation using the Vasculitis Damage Index (VDI) [19]; (vi) time interval from induction until complete remission (months); (vii) the estimated glomerular filtration rate (eGFR) at presentation (using the 2021 CKD-EPI creatinine equation). Severe disease was defined by an eGFR <50 ml/min/1.73 m^2^ among patients with kidney involvement or diffuse pulmonary haemorrhage at presentation [21].

During the follow-up, we also recorded the RTX maintenance scheme, the number of RTX cycles, and the cumulative RTX dose, as well the chemoprophylaxis against *Pneumocystis jirovecii*. In case of relapse, we recorded the daily prednisolone equivalent dose, organ involvement, BVASv3 and therapeutic decisions (GC pulses or increment of oral dose, reinduction with RTX/CYC/RTX + CYC/other).

### Statistical analysis

Continuous variables are presented as a median (interquartile range, IQR) and categorical variables as an absolute number (%). Mann–Whitney and χ^2^/Fisher’s tests were used for a comparison of continuous and categorical variables, respectively. The incidence rate (IR) was calculated by dividing the number of events during the follow-up with the patient-years of follow-up.

Kaplan–Meier plots was used to visualize the relapse- and major relapse–free survival in the overall cohort, as well between subgroups. Log-rank was used to compare the survival curves, while we also checked for time-varying covariance. Subsequently, unadjusted and adjusted Cox proportional hazards models were used to examine for factors associated with the time-to-first-relapse (major/minor or major), presenting the hazard ratios (HRs) and the corresponding 95% CI. For multivariable analyses, the independent variables were not highly correlated so as to avoid multicollinearity (details in Supplementary Data S2, available at *Rheumatology* online). A sensitivity analysis was conducted, excluding those patients who were followed up for <24 months, those whose follow-up ended due to end of the study, end of maintenance, non-compliance, delayed infusion or LFU.

Statistical significance was considered for *P*-values of <0.05. Analyses were performed using STATA 13.0 (StataCorp. 2013. Stata: Release 13. Statistical Software. College Station, TX: StataCorp LP), and graphs were constructed through the SPSS 24.0 (SPSS software, USA) software.

## Results

### Patient characteristics

Overall, 101 patients with GPA (69%) and MPA (31%) were included. Their patient, disease and treatment characteristics are presented in Table 1. The median age at RTX maintenance initiation was 63 years; 48% of patients were females, 54% were newly diagnosed and 93% were ANCA positive (52% c-/PR3-ANCA+, 41% p-/MPO-ANCA+).

**Table 1.** Patient, disease and treatment characteristics

Variable	Total *N* = 101
**Females, *n* (%)**	48 (48)
**Age at RTX maintenance initiation (years), median (IQR)**	63 (48–73)
**Disease duration (years), median (IQR)**	1.5 (0.6–4.1)
**Newly diagnosed, *n* (%)**	54 (53)
**AAV phenotype, *n* (%)**	
GPA	70 (69)
MPA	31 (31)
**ANCA serotype (ever)** ^a^ **, *n* (%), *n* = 100**	
c-/PR3-ANCA	52 (52)
p-/MPO-ANCA	41 (41)
Negative	7 (7)
**Characteristics at RTX maintenance initiation**	
**Induction to RTX-maintenance initiation (months), median (IQR)**	7 (6–12)
**Comorbidities** ^b^ **, *n* (%)**	
Arterial hypertension	50 (50)
Diabetes	16 (16)
Cardiovascular disease and/or stroke	23 (23)
Depression	15 (15)
Chronic obstructive pulmonary disease	4 (4)
Fracture	8 (8)
Malignancy	6 (6)
Peptic ulcer or other stomach problem	5 (5)
**Rheumatic Disease Comorbidity Index, median (IQR), *n* = 90**	1 (0–2)
**End-stage kidney disease, *n* (%)**	7 (7)
**Vasculitis damage index, median (IQR), *n* = 93**	1 (0–2)
**No. of previous relapses, *n* (%)**	
0	54 (54)
1	35 (35)
2	7 (7)
≥3	5 (5)
**IgG levels** (*n* = 54)	
Normal (≥700 mg/dl), *n*	42
Hypogammaglobulinemia (<700 mg/dl), *n*	12
**Characteristics at presentation**	
**Organ/System involvement, *n* (%)**	
Constitutional symptoms	53 (53)
Skin	11 (11)
Eyes/mucosal	8 (8)
ENT	29 (29)
Lung	62 (61)
Nervous	14 (14)
Cardiovascular	4 (4)
Kidney	55 (55)
eGFR^c^, median (IQR)	32 (11–87)
Haematuria (RBCs >10 hpf), *n* (%)	39 (71)
Protein >1+ stick urine, *n* (%)	32 (58)
Proteinuria (g/24 h), median (IQR)^d^	1.4 (0.6–2.5)
Rise in serum creatinine by >30% or fall in creatinine clearance by >25%, *n* (%)	31 (56)
Kidney biopsy, Berden classification, *n* (%)	*n* = 29
focal	11 (38)
crescentic	5 (17)
mixed	11 (38)
sclerotic	2 (7)
**BVAS version 3, median (IQR)**	10 (6–18)
**Haemodialysis, *n* (%)**	12 (12)
**Plasma exchange, *n* (%)**	9 (9)
**Induction treatment until complete remission, *n* (%)**	
RTX-containing regimens	
RTX alone	65 (64)
RTX + MTX/MMF^e^	5 (5)
Cumulative dose of RTX (g), median (IQR)	2 (2–3.5)
RTX+CYC	21 (21)
Cumulative dose of RTX (g), median (IQR)	2 (2–3.8)
Cumulative dose of CYC (g), median (IQR)	4.8 (2.1–6)
CYC monotherapy	10 (10)
Cumulative dose of CYC (g), median (IQR)	5.7 (4.4–6.7)
**No. of RTX-maintenance courses**	***n* = 135**
**RTX-maintenance dosing schemes (q 6 months), *n* (%)**	
500 mg	2 (3)
1000 mg	80 (59)
2000 mg (2 weeks apart)	27 (20)
variable^f^	26 (19)

a Among the ANCA-positive patients (*N* = 93), 64 (69%) tested positive for PR3-/MPO-ANCA through ELISA, while 16 (17%) had only p-/c-ANCA+ through indirect immunofluorescence. For 13 (14%) patients, the method of ANCA testing was not available.

b Present at RTX maintenance initiation.

c ml/min/1.73 m^2^.

d Available for 30/55 patients.

e Four patients received MTX and one MMF.

f Switch of RTX dose between cycles (500, 1000 or 2000 mg). RTX: rituximab; IQR: interquartile range; AAV: ANCA-associated vasculitis; GPA: granulomatosis with polyangiitis; MPA: microscopic polyangiitis; eGFR: estimated glomerular filtration rate; RBCs: red blood cells; hpf: high-power field; RTX: rituximab.

At presentation, the median BVASv3 was 10, while 61% of patients presented with lung, 29% with ΕΝΤ and 14% with neurologic manifestations. Almost half of the patients (55%) had kidney involvement, with a median eGFR at presentation of 32 ml/min/1.73 m^2^. Clinical manifestations of kidney involvement, as well data from the available kidney biopsies according to the Berden histopathological classification [22] are presented in Table 1.

Overall, 12% and 9% of patients underwent haemodialysis and PLEX, respectively. The induction regimens included (apart from GCs) RTX (64%), CYC (10%) or their combination (RTX+CYC: 21%) (Table 1). The median (IQR) time from induction until complete remission was 7 (6–12) months.

The overall median (IQR) follow-up time was 1.4 (0.6–3.3) years (294.5 patient-years), during which patients received a median (IQR) of 3 (2–6) RTX cycles and 4 (2–8) g of cumulative RTX dose. In total, 135 RTX maintenance courses were administered: 71 patients received one, 26 patients two and 4 patients three RTX courses, respectively. The RTX maintenance schemes used are shown in Table 1.

**Figure 1.**
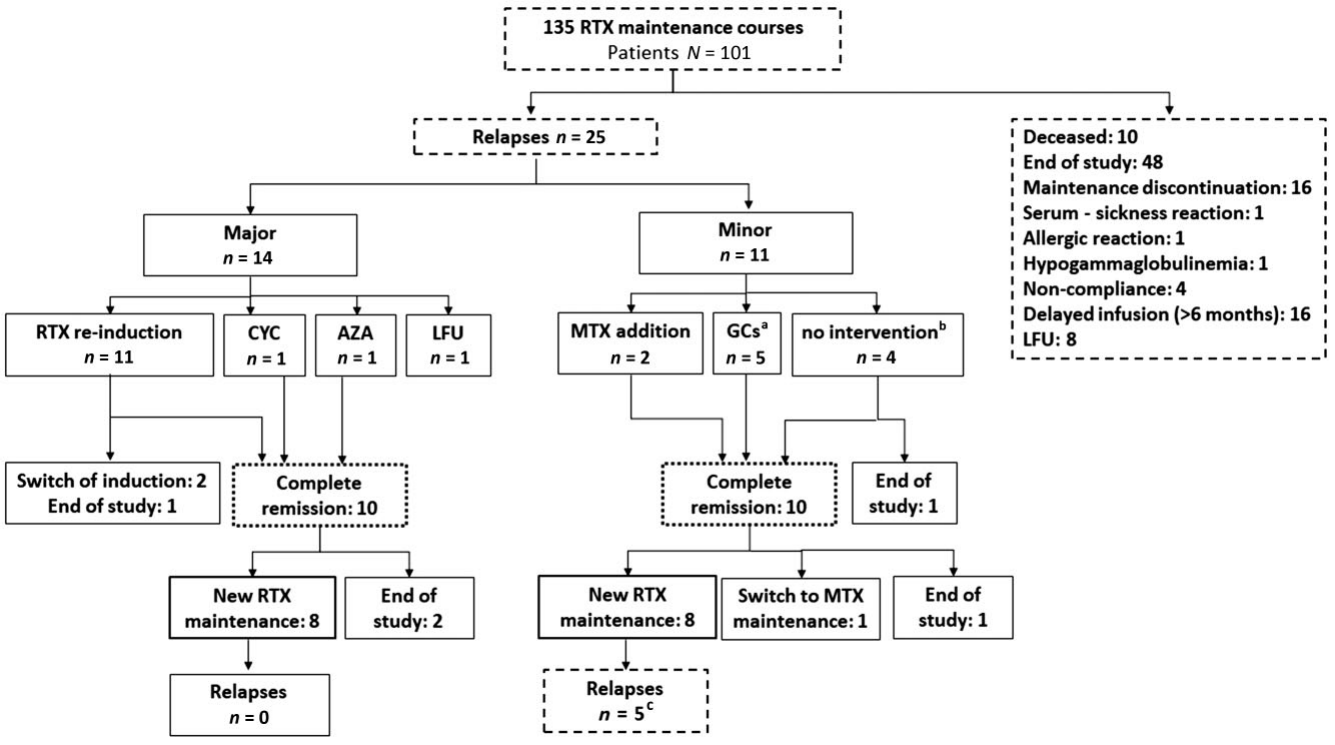
A flow-chart depicting the therapeutic decisions after relapses during RTX-maintenance therapy. ^a^I.m. GCs or increment of oral GCs dose. ^b^Continuation of RTX-maintenance therapy as was scheduled without additional interventions. ^c^Five relapses (three major and two minor) in five patients. Among the patients with major relapse, one patient achieved complete remission with RTX induction and then received a new RTX maintenance course, but the study ended before 6** **months of follow-up, while the other two patients had not achieved complete remission with RTX induction by the end of the study. Both patients with minor relapse were lost to follow-up (one after relapse and the other after achievement of complete remission with GCs). RTX: rituximab; LFU: lost to follow-up; GCs: glucocorticoids

Overall, 16 (12% of) RTX maintenance courses were discontinued due to the physician’s decision, 3 (2%) due to drug-related side effects (1 allergic reaction, 1 serum-sickness reaction and 1 severe hypogammaglobulinemia case), 8 (6%) due to LFU and 4 (3%) due to non-compliance (Fig. 1).

### Relapses during RTX maintenance

#### Incidence, timing and type of relapses

During follow-up (294.5 patient-years), we recorded 30 relapses in 24 patients (24%) with an IR of 10.2/100 patient-years (Table 2A). Most relapses (73%) occurred within the first 2 years after RTX maintenance initiation. The respective IR of relapses was 41.8, 15.6 and 3.7 per 100 patient-years within the first, second and ≥third year from RTX maintenance initiation.

**Table 2.** Overall and according to the year after initiation of RTX maintenance therapy incidence rates of (A) relapses and (B) serious infections

	Year of follow-up			
	Overall	1st	2nd	>3rd
**(A) Relapses**				
No. of events	**30**	**16**	**6**	**8**
No. of RTX courses	135	59	25	51
%	100%	53%	20%	27%
Patient-years	294.5	38.3	38.5	217.8
Incidence rate (per 100 patient-years)	**10.2**	**41.8**	**15.6**	**3.7**
**(B) Serious infections** ^a^				
No. of events	**17**	**8**	**1**	**8**
%	100%	76%	0%	24%
Patient-years	294.5	38.3	38.5	217.8
Incidence rate (per 100 patient-years)	**5.8**	**20.9**	**2.6**	**3.7**

a Excluding SARS-CoV-2–associated infections, which required hospitalization. RTX: rituximab; SARS-CoV-2: severe-acute-respiratory-syndrome-related coronavirus.

Among the 30 relapses, 17 (57%, 16 patients) were major, of which 9 (53%) involved the lungs, 3 (18%) ENT, 3 (18%) kidneys, 2 (12%) nerves, 2 (12%) eyes and/or mucosal and 1 (6%) the cardiovascular system. Minor relapses [*n* = 13 (43%), 11 patients] were mainly manifested as arthritis/arthralgia (77%) and 2 (15%) as ENT disease (Supplementary Table S1, available at *Rheumatology* online).

The median (IQR) BVASv3 at major and minor relapses was 6 (4–6.5) and 1 (1–1.5), respectively. The median (IQR) daily prednisolone dose at the time of relapse was 0 (0–5) mg.

#### Management of relapses

For major relapses (*n* = 14), most patients received RTX reinduction (*n* = 11), two other therapies (CYC *n* = 1, AZA *n* = 1) and one was LFU (Fig. 1). Of them, complete remission was achieved in 8 (73%), 1 (100%) and 1 (100%) patients, respectively. Subsequently, 6, 1 and 1 patients, respectively, received a new RTX maintenance course with no subsequent relapses.

Minor relapses (*n* = 11) were managed with the addition of GCs (*n* = 5) or MTX (*n* = 2), while four patients continued the RTX maintenance without interventions. Complete remission was achieved in five (100%), two (100%) and three (74%) patients, respectively. Thereafter, four, two and two patients, respectively, received a new RTX maintenance course, of whom 2/4 (50%), 2/2 (100%) and 1/2 (50%), respectively, relapsed. Among these five relapses, three were major and two minor. More details are presented in Fig. 1.

#### Factors associated with relapses

Patients who relapsed (*n* = 24), compared with those who did not (*n* = 77, Supplementary Table S2, available at *Rheumatology* online) less frequently had kidney involvement (33% *vs* 61%, *P = *0.014). There was no significant difference regarding the type of induction treatment, disease activity, ANCA serotype (c-/PR3-ANCA *vs* p-/MPO-ANCA) or the RTX maintenance dosing scheme (*P = *0.431).

**Figure 2.**
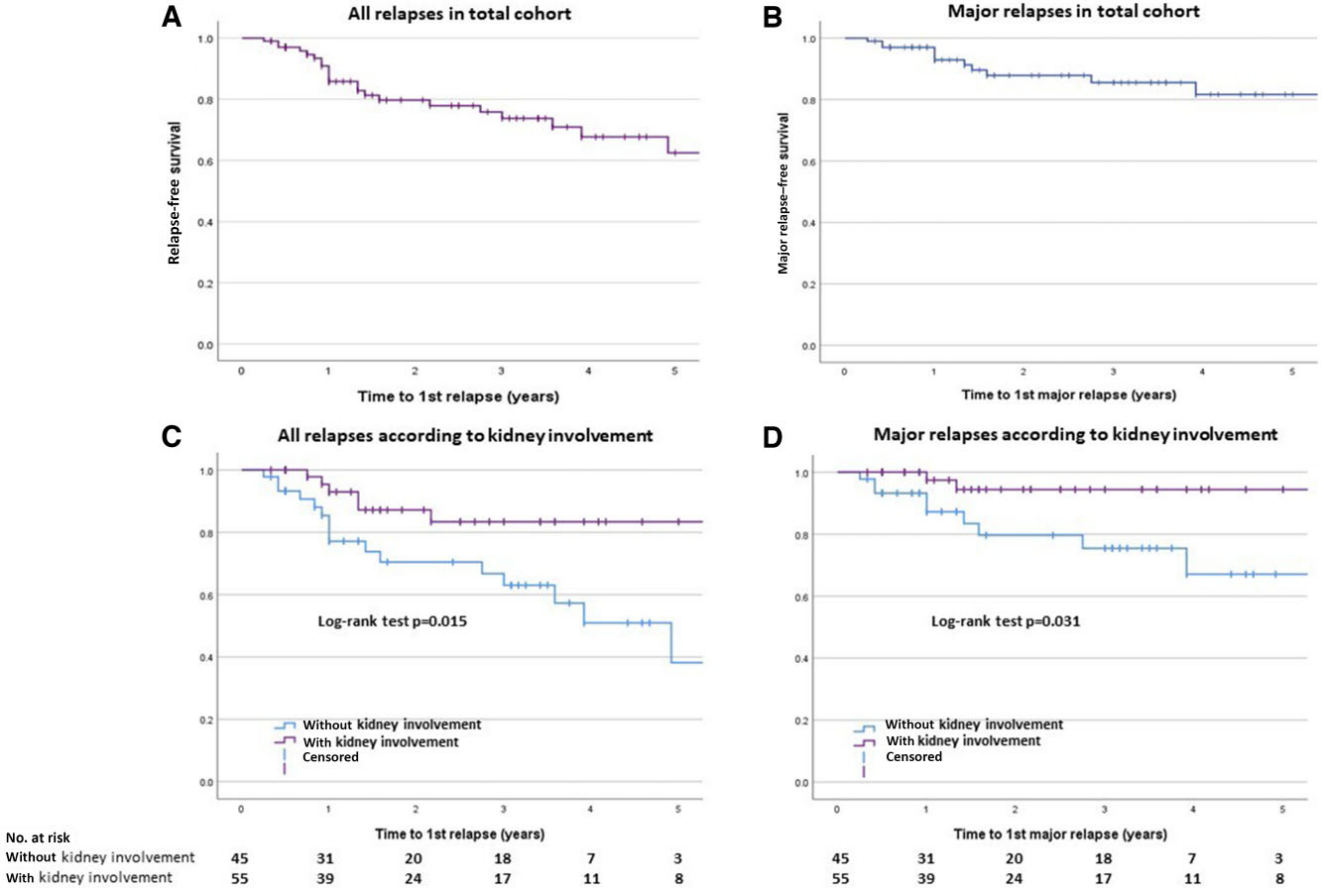
Relapse (major or minor)- and major relapse–free survival: (A) and (B) in the total cohort, and (C) and (D) according to kidney involvement

At year-2, the overall and major relapse-free survival probability was 0.80 (95% CI 0.69–0.88) and 0.88 (95% CI 0.78–0.94), respectively (Fig. 2). Relapse-free survival was significantly longer in patients with kidney involvement compared with those without (*P = *0.015, Fig. 2). Also, we observed a trend for longer relapse-free survival in patients with severe disease (*vs* non-severe; *P = *0.288, Supplementary Fig. S2, available at *Rheumatology* online), as well in those who received RTX + CYC combination for induction of remission (vs RTX or CYC monotherapy; *P = *0.064, Supplementary Fig. S4, available at Rheumatology online). No difference was observed in relapse risk between GPA and MPA, or between c-/PR3-ANCA– and p-/MPO-ANCA–positive patients, even after examining for time-varying covariance (Supplementary Fig. S3, available at *Rheumatology* online).

By Cox regression (Table 3), kidney involvement [adjusted HR (aHR): 0.20; 95% CI 0.06–0.74, *P = *0.016], prior induction with RTX+CYC combination (*vs* RTX monotherapy: aHR 0.02; 95% CI 0.001–0.43, *P = *0.012) and shorter time interval until complete remission (aHR 1.07; 95% CI 1.01–1.14, *P = *0.23) were associated with a lower relapse risk. For major relapses, only kidney involvement (aHR 0.10; 95% CI 0.01–0.72, *P = *0.022) was significantly associated with a lower relapse risk.

**Table 3.** Cox regression analysis for factors associated with the risk of relapse (major/minor or major) in AAV patients during RTX-maintenance therapy

	Major or minor relapse				Major relapse			
Variable	HR (95% CI)	*P*-value	**Adjusted HR** ^a^ **(95% CI)**	*P*-value	HR (95% CI)	*P*-value	**Adjusted HR** ^b^ **(95% CI)**	*P*-value
Females *vs* males	1.01 (0.44–2.30)	0.974	0.62 (0.23–1.68)	0.350	0.63 (0.20–2.00)	0.439	0.29 (0.07–1.23)	0.094
Age at RTX maintenance initiation (per year)	0.99 (0.96–1.01)	0.530	0.96 (0.93–1.00)	0.079	0.98 (0.94–1.02)	0.421	0.95 (0.91–1.00)	0.085
Newly diagnosed *vs* Relapsed	0.92 (0.40–2.10)	0.850	2.70 (0.65–11.13)	0.168	0.70 (0.22–2.24)	0.559	2.99 (0.36–24.85)	0.309
No. of previous relapses (per relapse)	**1.39 (1.01**–**1.91)**	**0.041**			**1.66 (1.13**–**2.43)**	**0.009**		
GPA *vs* MPA	1.16 (0.43–3.14)	0.766			1.67 (0.36–7.66)	0.508		
ANCA serotype (ever)								
c-/PR3 ANCA	Reference	–	Reference	–	Reference	–	Reference	–
p-/MPO ANCA	1.07 (0.45–2.54)	0.862	2.00 (0.67–5.98)	0.212	0.74 (0.22–2.49)	0.631	1.55 (0.30–7.80)	0.593
Negative ANCA	0.80 (0.10–6.21)	0.837	0.51 (0.05–5.36)	0.580	–	–	–	–
IgG ≥700 mg/dl at RTX maintenance initiation (yes/no)^c^	1.66 (0.19–13.82)	0.639			0.52 (0.04–5.82)	0.601		
PLEX and/or haemodialysis at presentation (yes *vs* no)	0.30 (0.04–2.28)	0.249				1.000		
BVASv3 at presentation (per point)	0.96 (0.91–1.02)	0.226			0.96 (0.89–1.04)	0.419		
Induction treatment until complete remission								
RTX^c^	Reference	–	Reference	–	Reference	–	Reference	–
CYC	1.48 (0.34–6.44)	0.599	2.41 (0.45–12.70)	0.300	1.38 (0.17–11.07)	0.759	2.24 (0.20–24.18)	0.504
**RTX+CYC**	**0.14 (0.01**–**1.06)**	**0.058**	**0.02 (0.001**–**0.43)**	**0.012**	–	–	–	–
**Kidney involvement (yes/no)**	**0.35 (0.14–0.85)**	**0.021**	**0.20 (0.06–0.74)**	**0.016**	**0.26 (0.07**–**0.97)**	**0.046**	**0.10 (0.01–0.72)**	**0.022**
Lung involvement (yes/no)	0.97 (0.41–2.26)	0.950	0.75 (0.27–2.11)	0.597	1.23 (0.36–4.14)	0.733	1.01 (0.23–4.84)	0.927
ENT involvement (yes/no)	1.27 (0.51–3.13)	0.597	0.73 (0.23–2.25)	0.592	1.49 (0.43–5.11)	0.525	0.71 (0.12–4.16)	0.709
**Time from induction to complete remission (per month)**	**1.01 (0.97**–**1.06)**	**0.406**	**1.07 (1.01**–**1.14)**	**0.023**	0.99 (0.93–1.07)	0.985	1.07 (0.97–1.18)	0.166

In adjusted model we included the age (per 1 year increment), sex (female *vs* male), onset of disease (newly diagnosed *vs* relapsed), ANCA serotype [ordinal; BVASv3: BVAS version 3; PR3/c-ANCA positivity (reference) *vs* MPO/p-ANCA positivity *vs* ANCA negativity], kidney, lung and ENT involvement (yes *vs* no), induction treatment received until complete remission [ordinal; RTX monotherapy or combined with MTX/MMF (reference) *vs* RTX+CYC *vs* CYC monotherapy] and the time interval from induction to RTX maintenance initiation (per 1 month increment). Statistically significant results (*P* < 0.05) are presented in bold.

a Observations included in the adjusted model *N* = 99.

b Observations included in the adjusted model *N* = 99.

c Available for 54/101 patients. HR: hazard ratio; RTX: rituximab; GPA: granulomatosis with polyangiitis; MPA: microscopic polyangiitis; PLEX: plasma exchange.

In sensitivity analysis, the same variables remained significantly associated with the risk of relapses, while age was also negatively associated with the relapse risk (Supplementary Table S3, available at *Rheumatology* online).

### Serious adverse events during RTX maintenance

#### Serious infections

We recorded 17 SIs in 14 (14%) patients at an IR of 5.8/100 patient-years (Table 2B). The majority were of respiratory tract origin (41%), followed by gastrointestinal (18%), herpes zoster (18%), urinary (12%) and bloodstream (12%) infections (Table 4). Most SIs (76%) occurred within the first year after RTX maintenance initiation.

**Table 4.** Serious adverse events during RTX maintenance therapy

Serious adverse events	No. of events (%)	IR/100 PY
**Serious infections (SI)** ^a^	**17**	**5.8**
Type of SI, *n* (%)		
Respiratory	7 (41)	
Urinary	2 (12)	
Bloodstream	2 (12)	
Gastrointestinal	3 (18)	
Zoster	3 (18)	
**COVID-19 associated hospitalizations**	**11**	**3.7**
Outcome, *n* (%)		
Recovery	8 (73)	
Death	3 (27)	
**Cardiovascular events (CVEs)**	**6**	**2.0**
Type of CVE, *n* (%)		
Coronary revascularization	2 (33)	
Peripheral angioplasty	1 (17)	
Stroke	3 (50)	
**Malignancies**	**4**	**1.4**
Type of malignancy, *n* (%)		
Lung	1 (25)	
Bladder	1 (25)	
Gastrointestinal	1 (25)	
Non-Hodgkin lymphoma	1 (25)	
**Deaths**	**10**	**3.4**
Cause of death, *n* (%)		
Infection	6 (60)	
Cardiovascular	3 (30)	
Malignancy	1 (10)	

a Excluding SARS-COV2–associated infections requiring hospitalization. IR: incidence rate; PY: patient years; SARS-CoV-2: severe-acute-respiratory-syndrome-related coronavirus.

The respective IR was 20.9, 2.6 and 3.7 per 100 patient-years within the first, second and ≥third year from RTX maintenance initiation (Table 2B). In 71% (12/17) of these cases, patients were receiving chemoprophylaxis against *Pneumocystis jirovecii* at the time of SI development.

#### Other serious adverse events

Other SAEs (Table 4) included hospitalizations for COVID-19 (*n* = 11, IR 3.7/100 patient-years) among whom 27% of patients died, CVEs (*n* = 6, IR 2/100 patient-years) and malignancies (*n* = 4, IR 1.4/100 patient-years). Ten patients died (10%, IR 3.4/100 patient-years), and the main cause of death was infections (60%), followed by CVEs (30%) and malignancies (10%).

Finally, among patients with available IgG data during the follow-up (*n* = 53), 41 (77%) had normal IgG levels at RTX maintenance initiation. Among these, 8 (∼20%) patients developed hypogammaglobulinemia (6 mild, 1 moderate and 1 severe), a median of 18 months after RTX maintenance initiation. None of them received IgG replacement therapy. There was no significant difference in SI rate between those who developed hypogammaglobulinemia (2/8, 25%) and those who did not (6/33, 18%, *P = *0.642).

## Discussion

In this large, real-life study of GPA/MPA patients who received RTX maintenance therapy, relapses occurred in about one-quarter of patients, mostly within the first 2 years of treatment. Kidney involvement, prior induction with RTX + CYC combination and earlier complete remission were associated with a decreased relapse risk.

In our cohort, 24% of GPA/MPA patients relapsed (IR: 10.2/100 patient-years), mainly in the first 2 years from RTX maintenance initiation (76%). At year 2, the relapse rate was 20%, which is slightly higher than that in core RTX maintenance trials (10–16%) [12, 23–25]. In the MAINRITSAN and MAINRITSAN2 trials, the relapse rates up to month 28 were 10% and 16%, respectively [23, 24], whereas in the RITAZAREM trial (relapsed GPA/MPA patients treated with RTX 1000 mg every 4 months) the respective rate was 15% [25]. Similarly, in small observational AAV studies with patients who had received RTX induction followed by RTX maintenance, 12–13% of patients relapsed during RTX maintenance [12, 15]. However, the above studies differ in terms of relapse and remission definitions, and so comparisons should be made cautiously.

Most relapses were major, involving the lungs, followed by the kidney and ENT. In the majority of cases, these were managed with RTX reinduction followed by RTX maintenance, without subsequent relapses. The role of reinduction with RTX has been evaluated in the RITAZAREM trial of 170 relapsing GPA/MPA patients (who had previously achieved remission with various agents, including RTX) who received reinduction therapy with RTX followed by RTX or AZA remission maintenance therapy. RTX was superior to AZA in preventing relapses during the maintenance phase (15% *vs* 38%, respectively) [25, 26].

Patients who experienced minor relapses (mainly arthritis/arthralgias) were managed either with the addition of GCs/MTX or continued the RTX maintenance scheme. Almost all patients achieved complete remission; however, about two-thirds of them relapsed during RTX maintenance therapy. Miloslavsky *et al.*, in analysing a subgroup of the RAVE trial with 44 GPA/MPA patients, also observed that the treatment of non-severe relapses with an increase in GCs restored remission in most of the cases (80%), but that the majority (70%) experienced a subsequent relapse shortly after, during the following AZA maintenance period [27, 28]. Experimental studies are needed to support the optimal strategy for the management of minor relapses.

One of the major goals of our study was to identify baseline predictors for relapse in RTX-treated patients. We found that the overall and major relapse risk was significantly lower in patients with kidney involvement. Accordingly, previous randomized controlled trials (RCTs) [5] and observational studies [29, 30] have identified that preserved kidney function and/or absence of kidney involvement were negative predictors of relapse. Indeed, once AAV patients with kidney disease achieve complete remission, they have a much lower relapse risk (usually patients with MPA/anti-MPO+), compared with those with lung disease (usually patients with GPA/anti-PR3+) [31]. So far, there is no clear explanation for this. These patients may receive more aggressive treatment (RTX, CYC or their combination with or without PLEX), which may lead to complete remission and reduced relapse risk. However, in our study, kidney involvement was significantly associated with a lower relapse risk independently of the induction treatment received.

Induction with RTX + CYC combination (*vs* RTX monotherapy) was also found to be independently associated with a lower risk of relapses. To date, this combination has been tested only in the RITUXIVAS trial [32], an open-label RCT in which AAV patients with renal involvement received GCs plus either RTX with two CYC pulses (*n* = 33) or i.v. CYC for 3–6 months followed by AZA (*n* = 11). The combined regimen was not found to be superior to the CYC regimen, and had similar rates of sustained remission. In real-life, a small retrospective cohort of 62 AAV patients who had received induction with either RTX + low-dose CYC or RTX alone, both followed by patient-tailored RTX maintenance, showed that the major relapse rates at year 2, but not after 5 years, were significantly lower in the combination group (3% *vs* 24%, *P = *0.032) [33]. Another retrospective cohort of 129 AAV patients, although without a control group, showed that the combination of RTX with a 2-month course of oral, low-dose CYC and a rapid GC tapering was efficacious and well tolerated [34].

We also observed that the achievement of earlier complete remission after induction was associated with a lower risk of subsequent relapses on RTX maintenance. Interestingly, a pooled analysis of four inception EUVAS trials, with different induction regimens, showed that patients who achieved remission by month 3 and who had maintained it at month 6 had a lower mortality risk [35]. More data are needed to clarify the impact of earlier disease remission on short- and long-term outcomes (relapses, end-stage kidney disease, morbidity, mortality).

Previous retrospective studies have shown that PR3-ANCA or GPA patients had higher relapse risk compared with MPO-ANCA or MPA patients, respectively [8, 12]. In agreement with the MAINRITSAN and RITAZAREM trials [5, 25], we did not find a significant association between ANCA serotype or clinical phenotype with the risk of relapse. Of note, a post-hoc analysis of the RAVE trial showed that PR3-ANCA patients respond better to RTX than to CYC/AZA induction therapy, suggesting that the predictive factors of relapse may differ in RTX-treated patients [36]. Finally, this was also the case for ENT involvement at presentation, contrary to the findings of the MAINRITSAN trials in which it was found to be associated with relapses [5].

The majority of our participants received higher RTX doses than recommended (Table 1) [11], reflecting real-world practice over the past years. However, we found that relapses were not significantly associated with the RTX maintenance dosing scheme. RCTs comparing different RTX-maintenance doses are needed to answer this important question.

Regarding the real-world safety of RTX maintenance, we observed an SI rate of 5.8/100 patient-years, which was consistent with previous RTX maintenance studies, with most of them being of respiratory tract origin [37] and having occurred in the first year after RTX maintenance initiation [38, 39]. At year 2, we found an IR of 11.7/100 patient-years. Furthermore, at month 28, in the MAINRITSAN trial, in which all patients had received CYC induction, the IR was 8.3/100 patient-years [23], while in the MAINRITSAN2 trial, in which patients had received CYC or RTX induction, the IR was 9.5/100 patient-years [24]. Although SIs remain an issue for AAV patients, we have previously demonstrated no difference in SI rates between RTX and other maintenance regimens [38], while a recent meta-analysis of RCTs and observational studies indicated a very low infection-related mortality rate (1.29%) during RTX maintenance [37].

To the best our knowledge, this is the first observational AAV study with longitudinal data for COVID-19 outcomes during RTX maintenance therapy. We observed an increased rate of COVID-19 hospitalizations during the late study period, with a considerable mortality rate. In the MAINTAINCAVAS trial, an open-label RCT that compared two remission maintenance strategies in 117 AAV patients, COVID-19 hospitalizations occurred in 7 patients (6%), of whom one died, during a median follow-up of 4.1 years [40]. In people with rheumatic diseases, RTX has been associated with worse COVID-19 outcomes [41]. In a cross-sectional study of 180 AAV patients on induction/maintenance with diverse regimens, COVID-19–related mortality was higher than in the general population, while the risk of COVID-19 infection and its severity did not differ among the various treatment groups [42]. However, data from nationwide studies indicate a decline in COVID-19 hospitalizations and deaths among patients with rheumatic diseases, with vaccination and the dominance of milder virus variants being key contributors [43].

The mortality rate during follow-up was 10%, with SIs being the leading cause of death, followed by CVEs and malignancies. Regardless of therapy, it seems that AAVs are associated with a high risk of CVEs [44]. While RTX has been found to have beneficial effects on endothelial function in patients with RA [45], evidence for cardiovascular outcomes in RTX-treated AAV patients is lacking [46]. As for malignancies, the data indicate that AAV patients treated with RTX have no increased risk compared with the general population [47].

Hypogammaglobulinemia was developed in ∼20% of patients with normal IgG levels at RTX maintenance initiation, but there was no difference in the SI rate compared with those without hypogammaglobulinemia. In the RITAZAREM trial, the incidence of hypogammaglobulinemia among RTX-treated patients was 42% and was related to increased glucocorticoid exposure and low baseline IgG [25].

Limitations of our study include its retrospective design, the heterogeneity in RTX dosing and in the protocols for CYC induction, reflecting the diverse scenarios encountered in real life and the changes in the management of AAV over the last years. Additionally, we had missing values for IgG and ANCA levels, while methods for ANCA testing varied between centres, which highlight the absence of a common clinical practice. Also, censored cases influenced the study sample during the follow-up; thus, we may have overestimated relapse rates at year 2. Finally, data for GC cumulative dose, the tapering schedule for GCs, and the patients’ vaccination histories were not available.

Our study has important strengths. First, it had a relatively large sample size and long-term follow-up in a real-world setting. In addition, we utilized rather strict definitions for remission and relapses compared with previous studies. Thus, for complete remission we incorporated the GC dosage (prednisolone ≤7.5 mg/day) in combination with BVASv3 = 0, compared with the RITAZAREM trial [25], in which the cut-off prednisolone dose was ≤10mg/day, and the MAINRITSAN trial [23], in which the GC dose was not included. Finally, we present novel data regarding the incidence and outcomes of COVID-19 hospitalizations during RTX maintenance in AAV patients.

In conclusion, our study showed that real-life RTX maintenance therapy was efficacious, with relapses occurring in approximately one-quarter of patients. Most relapses occurred during the first 2 years of treatment and were usually managed successfully with RTX reinduction. Patients with kidney involvement, those who received RTX + CYC induction, and those achieving earlier complete remission were at lower relapse risk. SIs remain a concern, especially during the first year of RTX maintenance therapy, constituting the leading cause of death in this population. Furthermore, we noted an increased rate of COVID-19–associated hospitalizations during the late study period.

Our findings support the efficacy and safety of RTX as a long-term maintenance agent for GPA/MPA patients after complete remission, raising an interesting question whether more efficacious induction schemes and earlier complete remission are associated with a lower long-term relapse risk. Infections always remain a concern, emphasizing the need for close monitoring and strategies to prevent infectious complications. The question remains of whether the available measures are adequate or whether there are additional factors that could contribute to a further limitation of infections in RTX-treated AAV patients.

## Supplementary Material

keae409_Supplementary_Data

## Data Availability

The datasets used and/or analysed for this study are available from the corresponding author upon request.
