# Supplementary material for: Relapses and serious adverse events during rituximab maintenance therapy in ANCA-associated vasculitis: a multicentre retrospective study
Source: Rheumatology (Oxford). 2024 Aug 6;64(4):1989–98. doi: 10.1093/rheumatology/keae409 (PMC11962940; doi:10.1093/rheumatology/keae409)
Supplement: keae409_Supplementary_Data [file keae409_supplementary_data.docx]

**Supplementary Data S1. Definitions for comorbidities**

Definitions for comorbidities: hypertension (systolic blood pressure ≥140mmHg and/or diastolic blood pres­sure ≥90mmHg in two measurements and/or administration of antihypertensives), diabetes (fasting blood sugar ≥126mg/dl and/or anti-diabetic drugs), history of cardiovascular disease and/or stroke, depression (diagnosed by a psy­chiatrist), osteoporotic fractures (confirmed by radiography), peptic ulcer or other stomach problems (confirmed by a gastroenterologist), chronic obstructive pulmonary disease (confirmed by spirometry).

**Supplementary Data S2. Independent variables included in adjusted Cox regression analysis**

We included the age (per 1 year increment), the sex (female vs male), the onset of disease (newly-diagnosed vs relapsed), the ANCA type (ordinal; PR3/c-ANCA positivity [reference] vs MPO/p-ANCA positivity vs ANCA negativity), the kidney, lung, and ear-throat-nose (ENT) involvement (yes vs no), the induction treatment received until complete-remission (ordinal; RTX monotherapy or combined with MTX/MMF [reference] vs RTX+CYC vs CYC monotherapy) and the time interval from induction to RTX-maintenance initiation (per 1 month increment).

**Supplementary Table S1. Organ involvement at relapse.**

|  | **Major**  **n=17** | **Minor**  **n=13** |
| --- | --- | --- |
| **Joints** | 7 (41%) | **10 (77%)** |
| **Eyes/mucosal** | 2 (12%) | - |
| **Ear, nose, throat** | 3 (18%) | 2 (15%) |
| **Lung** | **9 (53%)** | - |
| **Kidney** | 3 (18%) | 1^*^ (8%) |
| **Nervous** | 2 (12%) | - |
| **Cardiovascular** | 1 (6%) | - |

^*^This patient had only proteinuria and received upon physician’s decision 1g methylprednisolone as minor relapse.

**Supplementary Table S2. Comparison between patients who relapsed and those who did not during RTX-maintenance therapy.**

| **Variable** | **Relapsed**  **(n=24)** | **Non-relapsed**  **(n=77)** | **p-value** |
| --- | --- | --- | --- |
| Females, n (%) | 12 (50) | 36 (47) | 0.781 |
| Age at RTX-maintenance initiation, median (IQR) | 57 (49-72) | 64 (48-73) | 0.465 |
| Disease duration (years), median (IQR) | 1.7 (0.8-4.8) | 1.5 (0.6-3.6) | 0.525 |
| Follow-up (years), median (IQR) | 1.1 (0.8-2.7) | 1.8 (0.8-3.9) | 0.175 |
| Newly diagnosed, n (%) | 11 (46) | 43 (56) | 0.391 |
| n of previous relapses (per relapse) | 1 (0-1) | 0 (0-1) | 0.200 |
| GPA (vs MPA diagnosis), n (%) | 19 (79) | 51 (66) | 0.230 |
| C-/PR3-ANCA (vs P-/MPO-ANCA, ever) | 13 (54) | 39 (51) | 0.731 |
| Rheumatic Disease Comorbidity Index, median (IQR) | 1 (0-1.5) | 1 (0-2) | 0.125 |
| Vasculitis damage index, median (IQR) | 1 (0-2) | 1 (0-2) | 0.806 |
| Concomitant drugs for maintenance, n (%) | 3 (12.5) | 6 (8) | 0.480 |
| PLEX and/or hemodialysis at presentation, n (%) | 1 (4) | 15 (19) | 0.073 |
| BVASv3 at presentation, median (IQR) | 6 (4-14) | 12 (6-18) | 0.075 |
| Induction treatment until complete-remission, n (%) |  |  |  |
| RTX^§^ | 21 (88) | 49 (64) | 0.056 |
| CYC | 2 (8) | 8 (10) | - |
| RTX+CYC | 1 (4) | 20 (26) | - |
| Cumulative induction dose of RTX (gm), median (IQR) | 2 (2-4) | 2 (2-2.8) | 0.585 |
| Organ involvement, n (%) |  |  |  |
| Constitutional symptoms | 12 (50) | 41 (53) | 0.736 |
| Skin | 1 (4) | 10 (13) | 0.220 |
| Eyes/mucosal | 3 (12) | 5 (6) | 0.351 |
| ENT | 7 (29) | 22 (29) | 0.984 |
| Lung | 14 (61) | 48 (62) | 0.671 |
| **Kidney** | **8 (33)** | **47 (61)** | **0.014** |
| Nervous | 4 (17) | 10 (13) | 0.666 |
| Cardiovascular | 2 (8) | 2 (3) | 0.214 |
| Time from induction to complete-remission (months), median (IQR) | 7.5 (6.0-13.0) | 7.0 (6.0-12.0) | 0.804 |
| RTX-maintenance therapy |  |  |  |
| n of RTX cycles for maintenance, median (IQR) | 3 (2-5) | 4 (2-7) | 0.254 |
| Cumulative RTX dose for maintenance (gm), median (IQR) | 4.0 (2.0-6.5) | 5.0 (2.0-8.0) | 0.831 |

^§^RTX ± MTX/MMF; 65 patients received RTX monotherapy, 4 received RTX+MTX and 1 patient received RTX+MMF.

RTX; Rituximab, IQR; Interquartile range, n; number, MPA; microscopic polyangiitis, GPA; granulomatosis with polyangiitis, PLEX; plasma exchange, BVASv3; Birmingham Vasculitis Activity Score version 3, CYC; cyclophosphamide, g; grams. Statistically significant results (p<0.05) are presented in bold.

|  | **Major or minor relapse** | | | |
| --- | --- | --- | --- | --- |
| **Variable** | **HR (95%CI)** | **p-value** | **Adjusted HR^b^ (95%CI)** | **p-value** |
| Females vs males | 0.98 (0.43-2.23) | 0.971 | 0.46 (0.16-1.28) | 0.141 |
| **Age at RTX-maintenance initiation (per year)** | 0.99 (0.96-1.02) | 0.721 | **0.95 (0.92-0.99)** | **0.041** |
| Newly diagnosed vs Relapsed | 0.93 (0.41-2.12) | 0.874 | 5.09 (0.82-31.37) | 0.079 |
| ANCA serotype (ever) |  |  |  |  |
| c-/PR3 ANCA | reference | - | reference | - |
| p-/MPO ANCA | 1.21 (0.51-2.85) | 0.659 | 2.43 (0.65-8.95) | 0.182 |
| Negative ANCA | 1.10 (0.14-8.46) | 0.925 | 4.88 (0.54-44.04) | 0.158 |
| Induction treatment leading to complete-remission |  |  |  |  |
| RTX^§^ | reference | - | reference | - |
| CYC | 1.43 (0.33-6.24) | 0.627 | 2.44 (0.44-13.27) | 0.301 |
| **RTX+CYC** | 0.15 (0.02-1.19) | 0.074 | **0.004 (0.0001-0.18)** | **0.00** |
| **Kidney involvement (yes/no)** | 0.38 (0.15-0.94) | 0.037 | **0.14 (0.03-0.69)** | **0.016** |
| Lung involvement (yes/no) | 0.86 (0.37-1.99) | 0.726 | 0.68 (0.19-2.36) | 0.544 |
| ENT involvement (yes/no) | 1.29 (0.52-3.17) | 0.578 | 0.45 (0.12-1.64) | 0.231 |
| **Time from induction to complete-remission (per month)** | 1.03 (0.99-1.08) | 0.075 | **1.11 (1.04-1.19)** | **0.002** |

**Supplementary Table S3. Sensitivity analysis^a^: Cox regression analysis for factors associated with the risk of relapse (major/minor) in AAV patients during RTX-maintenance therapy.**

^a^Sensitivity analysis (n=65 patients): Among patients who were followed-up for <24 months, we excluded those whose follow-up ended due to: end of study, end of maintenance, non- compliance with therapy, lost to follow-up or delayed infusion.

^b^Observations included in the adjusted model N=64/65.

HR; Hazard Ratio, CI; Confidence Interval, RTX; Rituximab, GPA; granulomatosis with polyangiitis, MPA; microscopic polyangiitis, ANCA; antineutrophil cytoplasmic antibody, IgG; Immunoglobulin G, PLEX; plasma exchange, BVASv3; Birmingham-Vasculitis Activity Score version-3, RTX; Rituximab, CYC; cyclophosphamide, ENT; ear-nose-throat, n; number, gm; grams.

**Supplementary Figure S1. Study design**

**
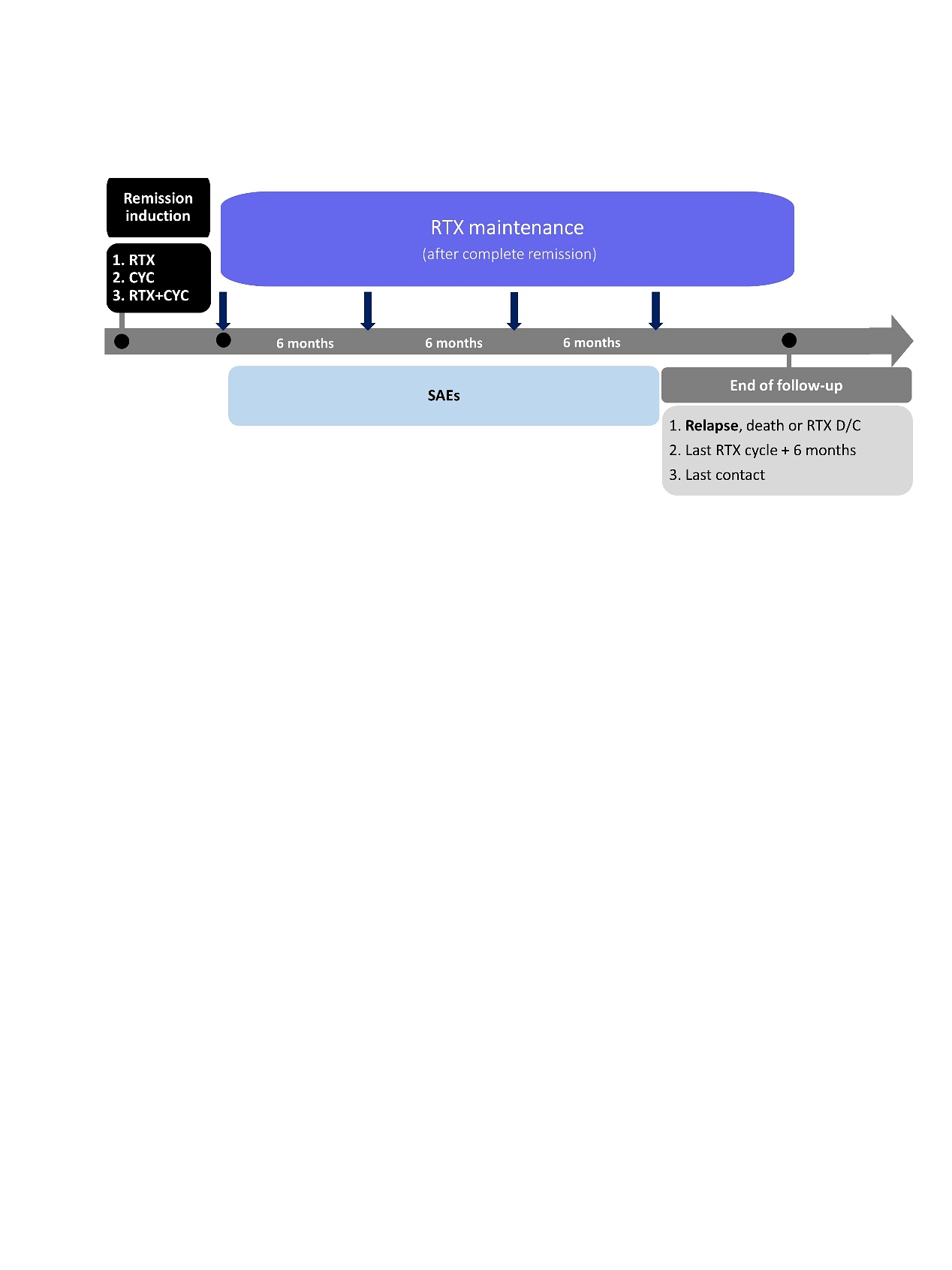
**

Complete-remission was defined as Birmingham-Vasculitis-Activity-Score version 3 =0 and prednisolone equivalent dose ≤7.5 mg/day.

RTX; Rituximab, CYC; Cyclophosphamide; SAEs; serious adverse events, D/C; discontinuation.

**Supplementary Figure S2. Relapse-free survival according to the presence of severe disease (renal involvement with eGFR <50 ml/min/1.73m² or diffuse pulmonary haemorrhage) at induction.**

**
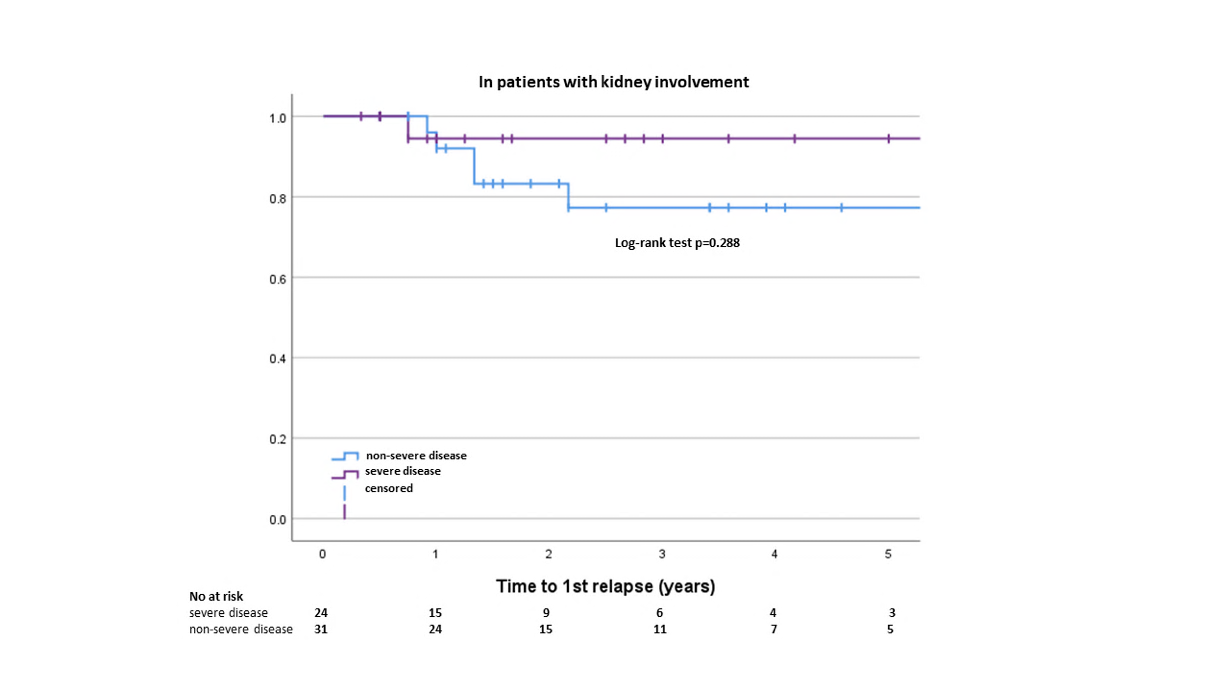
**

**Supplementary Figure S3. Relapse free survival according to: A. ANCA serotype and B. AAV phenotype.**


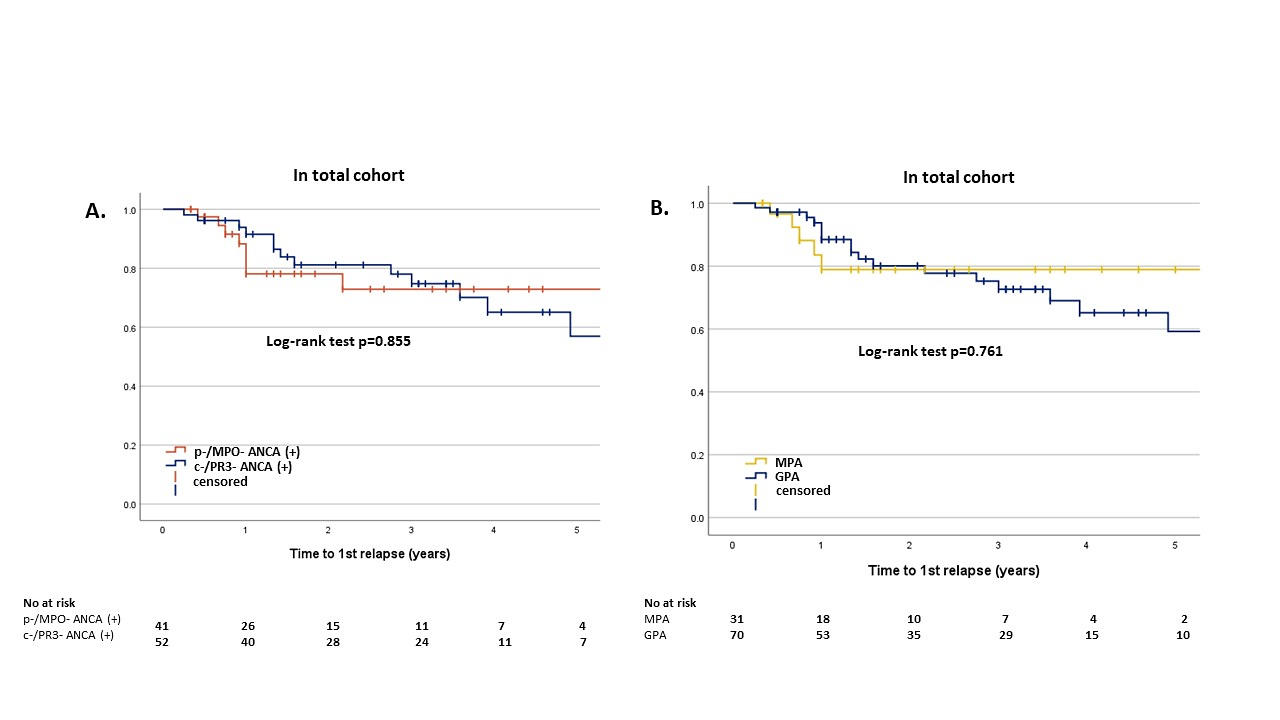


We also checked for time-varying covariance given the crossing curves, but this was not statistically significant, and the results of the adjusted cox models did not change.

MPA; microscopic polyangiitis, GPA; granulomatosis with polyangiitis.

**Supplementary Figure S4.** **Relapse-free survival according to treatment received for induction of remission.**


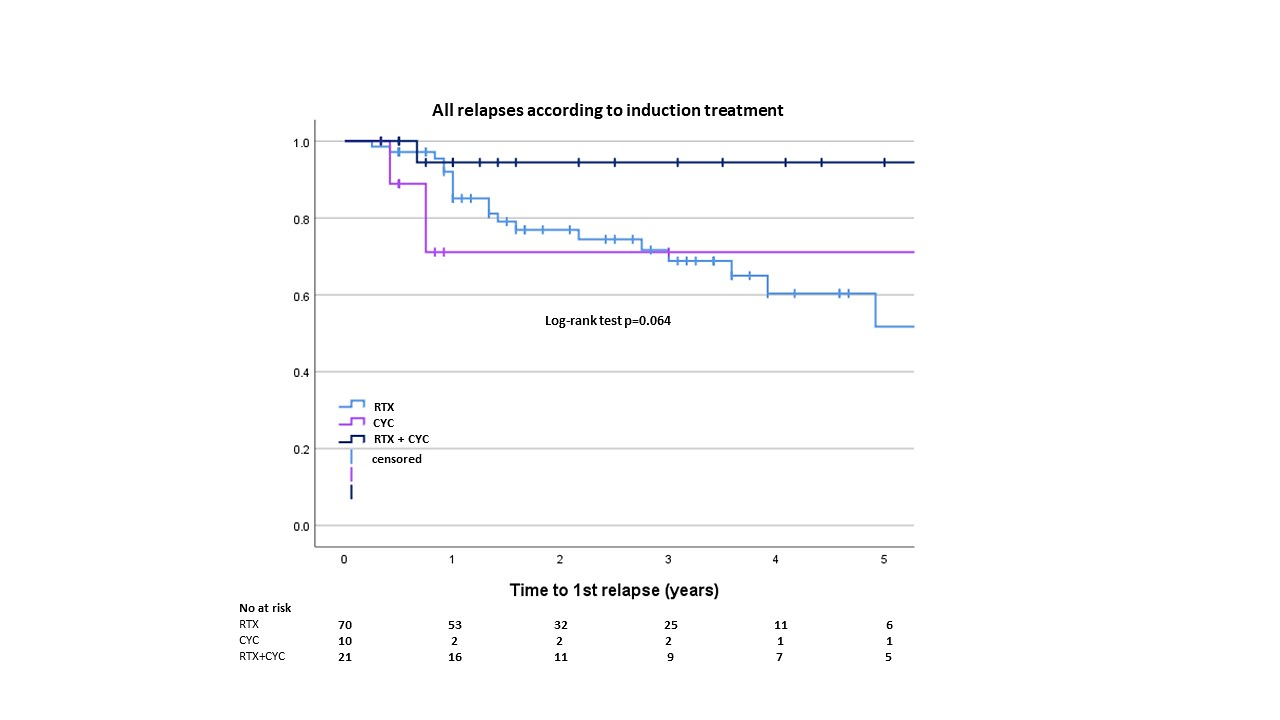


RTX; Rituximab, CYC; Cyclophosphamide.
